# Flowable Porcine Urinary Bladder Matrix in Wounds Complicated by Tunneling and Undermining: A Multicenter Prospective Study

**DOI:** 10.3390/jcm15166432

**Published:** 2026-08-20

**Authors:** Malachy E. Asuku, Hannah Baker, Saarah Mohammedi, Weiwei Xu, Marcie Jaffee, Jessica L. Evans, Yifei Dai, Claire Witherel, Yi Duan-Arnold, Kristy L. Hawley, Michael Cripps, Jeffrey W. Shupp, Alisha Oropallo

**Affiliations:** 1 Integra LifeSciences, Princeton, NJ 08540, USA; hannah.baker@integralife.com (H.B.); saarah.mohammedialseginy@integralife.com (S.M.); weiwei.xu@integralife.com (W.X.); marcie.jaffee@integralife.com (M.J.); jessica.evans@integralife.com (J.L.E.); yifei.dai@integralife.com (Y.D.); claire.witherel@integralife.com (C.W.); yi.arnold@integralife.com (Y.D.-A.); 2 Department of Surgery, Section of Trauma and Acute Care Surgery (TACS), University of Colorado Anschutz, Aurora, CO 80045, USA; kristy.hawley@cuanschutz.edu (K.L.H.); michael.cripps@cuanschutz.edu (M.C.); 3 MedStar Health, Washington, DC 20010, USA; jeffrey.w.shupp@medstar.net; 4 Northwell Health, Lake Success, NY 11042, USA; aoropallo@northwell.edu

**Keywords:** wound care, acellular tissue matrix, tunneled wounds, undermined wounds, urinary bladder matrices, chronic ulcers

## Abstract

**Objective:** This multicenter, prospective, single-arm study evaluated the safety and preliminary clinical performance of a flowable porcine urinary bladder matrix (UBM) particulate for managing tunneling and undermining features in complex wounds. These features are associated with delayed healing, chronic inflammation, and infection risk. Flowable UBM enables targeted delivery into wound tunnels and cavities. **Method:** Twenty-five subjects from 3 United States (U.S.) sites were enrolled, with 21 meeting protocol criteria for analysis (15 with undermining and 6 with tunneling wounds). Participants received flowable UBM applied directly to tunneling or undermining areas, along with additional UBM particulate or sheet forms applied to the wound surface. The primary endpoint was the percentage reduction in tunneling volume or undermining depth at 12 weeks, with secondary assessment of reduction in overall wound volume. Safety was evaluated through evaluation of device-related adverse events. **Results:** At 12 weeks, 90.5% of wounds demonstrated positive response, with mean ± standard deviation and median reduction in tunneling or undermining dimensions of 84.6 ± 29.3% and 100.0%, respectively. Complete resolution of these features occurred in 52.4% of wounds (53.3% in undermining and 50.0% in tunneling wounds) with higher closure rates in non-pressure wounds compared to pressure injuries. No device-related adverse events were reported. **Conclusions:** Overall, flowable UBM was well tolerated and associated with favorable clinical trajectories demonstrated by reduction in tunneling and undermining dimensions; however, comparative effectiveness cannot be inferred from this limited single-arm pilot study. Further controlled studies are needed to confirm comparative effectiveness and optimize clinical use.

## 1. Introduction

Complex wounds represent a substantial clinical and economic burden, accounting for an estimated 1–3% of healthcare expenditures in developed countries and approximately $25 billion annually in the United States [1]. Tunneling and undermining are common manifestations of complex wounds associated with persistent inflammation, increased infection risk, and delayed healing [2,3,4]. Exudate retained within tunneled and undermined spaces contains high concentrations of inflammatory mediators that further impair repair, necessitating effective management of dead space, drainage and compromised perfusion [5,6].

Cellular and acellular matrix-like products (CAMPs) have emerged as advanced therapies designed to restore extracellular matrix architecture and facilitate healing in complex wounds, including surgical wounds, open abdominal wounds, burns, traumatic injuries, and diabetic or venous ulcers [7,8,9,10]. Porcine urinary bladder matrix (UBM), an acellular extracellular matrix scaffold, has demonstrated clinical utility across multiple wound etiologies by supporting cellular infiltration, revascularization, and soft tissue remodeling [11,12,13,14,15,16,17,18,19,20,21,22,23,24,25,26,27,28,29,30,31]. A flowable UBM configuration utilizes a dual-syringe delivery system to facilitate controlled delivery of UBM particulate into wound tunnels, crevices and cavities, allowing targeted management of anatomically complex wounds. This approach may support granulation tissue formation and wound closure in areas that are difficult to access with conventional dressings. The present study reports the first prospective, multi-center evaluation of a flowable UBM (MicroMatrix^®^ Flex, Integra LifeSciences, Princeton, NJ, USA) in patients with wounds complicated by tunneling or undermining, assessing its safety and clinical performance when used in conjunction with UBM particulate and sheet formulations.

## 2. Materials and Methods

### 2.1. Study Design

This was a prospective, post-market single-arm pilot study designed to evaluate the preliminary safety and clinical performance of flowable UBM in the management of wounds exhibiting tunneling and undermining. The study was conducted at three U.S. medical centers located in Colorado, Maryland, and New York.

### 2.2. Ethical Approval and Patient Consent

The Biomedical Research Alliance of New York Investigational Review Board (IRB)approved the study protocol, which adhered to the 1975 Declaration of Helsinki and its subsequent amendments. The study was registered on ClinicalTrials.gov (NCT06629506). All subjects completed the informed consent process, including providing a signed and dated IRB-approved consent form that permitted the publication of deidentified study data.

### 2.3. Patient Screening and Eligibility

At the screening visit, patients were assessed for their eligibility according to the criteria outlined in Table 1. Eligible patients provided written informed consent. The provider performed a complete physical examination and comprehensive wound assessment. The patient’s complete medical history, wound history, and concomitant medications were also documented.

### 2.4. Study Assessments and Procedures

Study visits were conducted on day 1 (first treatment), week 2 (second treatment), week 4 (third treatment, optional at discretion of investigator), week 8 (follow-up), and week 12 (final follow-up).

On day 1, week 2, and, where applicable, week 4 treatment visits, wounds were debrided and treated using UBM-based products (MicroMatrix^®^ Flex, MicroMatrix^®^ UBM Particulate, and MicroMatrix^®^ UBM Particulate, Integra LifeSciences, Princeton, NJ, USA). Flowable UBM (MicroMatrix^®^ Flex) was applied to tunneling and undermining regions of the wound. UBM particulate (MicroMatrix^®^ UBM Particulate) and UBM sheets (Cytal^®^ Wound Matrix) were then applied to the wound surface according to the respective instructions for use. Product selections, sizing, and quantity were determined by wound characteristics and investigator judgement.

At the discretion of the surgical team, additional wound care procedures were implemented: dressing changes with moistened and/or dry gauze, mechanical debridement, negative pressure wound therapy (NPWT), hyperbaric oxygen therapy, topical antimicrobials, and systemic antibiotics.

During all post-screening visits, wound measurements were obtained after debridement. These included (1) wound dimensions using the Kundin method [32]—greatest width, greatest length, and depth—and (2) the maximum tunneling or undermining diameter and depth. Additional documentation included: standard wound care procedures performed, adverse events (AEs), wound photography, length of stay (as applicable), and concomitant medications and treatments.

Documentation at each treatment visit included: method of surgical debridement, device type(s) applied, use of NPWT, and device deficiencies where applicable.

For patients who received a split-thickness skin graft, additional assessments were conducted on the grafting date and at a 4-week follow-up visit. At these graft-related visits, wound dimensions were documented using the Kundin method along with standard study assessments.

### 2.5. Outcome Measures

The primary outcome was the relative rate of closure of a tunneling or undermining feature as determined through measurement of opening size and depth of managed wound at 12 weeks, relative to the baseline at first treatment visit.

Secondary outcomes included relative rate of closure of a tunneling or undermining aspect at each visit relative to first treatment visit; relative rate of surface wound closure as determined through measurement of volume (Kundin method) of the wound at each visit and per number of treatments received.

Safety outcomes included all device- and procedure-related AEs (wound infection, seroma and hematoma) and device deficiencies (allergic reaction and intolerance). The study also explored, as applicable, the area of skin graft utilized relative to the original wound size, skin graft take percentage at skin graft follow-up, and time to skin graft.

### 2.6. Outcome Calculations

The primary outcome of this study was the relative rate of closure of a tunneling or undermining feature as determined manually through measurement of opening size and depth of the managed wound at 12 weeks relative to the baseline at first visit. For tunneling wound aspects, volume was used to calculate the relative rate of closure; for undermined wound aspects, depth was used to calculate the relative rate of closure. For secondary outcomes, the relative rate of aspect closure was calculated for each visit, and the relative rate of overall (surface) wound closure was determined manually through measurement of volume using the Kundin method at each visit, relative to first treatment visit. Details and equations used in calculations are provided upon request from the corresponding author. A detailed description of the outcome calculation methods is provided in Appendix A.

### 2.7. Statistical Analysis

Statistical analyses were performed using SAS/STAT software, Version 9.4 (SAS Institute Inc., Cary, NC, USA). No formal hypothesis-driven sample size calculation was performed. The study was designed as an exploratory, post-market clinical investigation intended to generate preliminary safety and clinical performance data regarding management of tunneling and undermining wounds.

Descriptive statistics were used to analyze the study data. Continuous variables were summarized using the mean, standard deviation (S.D.), median, and range, as appropriate. Categorical variables were summarized as counts and percentages, with 95% confidence intervals reported where applicable.

### 2.8. Use of Artificial Intelligence (AI)

Microsoft 365 Copilot was used solely for spelling and language refinement during manuscript preparation. The authors assume full responsibility for the content of the final, reviewed, and edited article.

## 3. Results

### 3.1. Study Cohort and Baseline Characteristics

**Figure 1:**
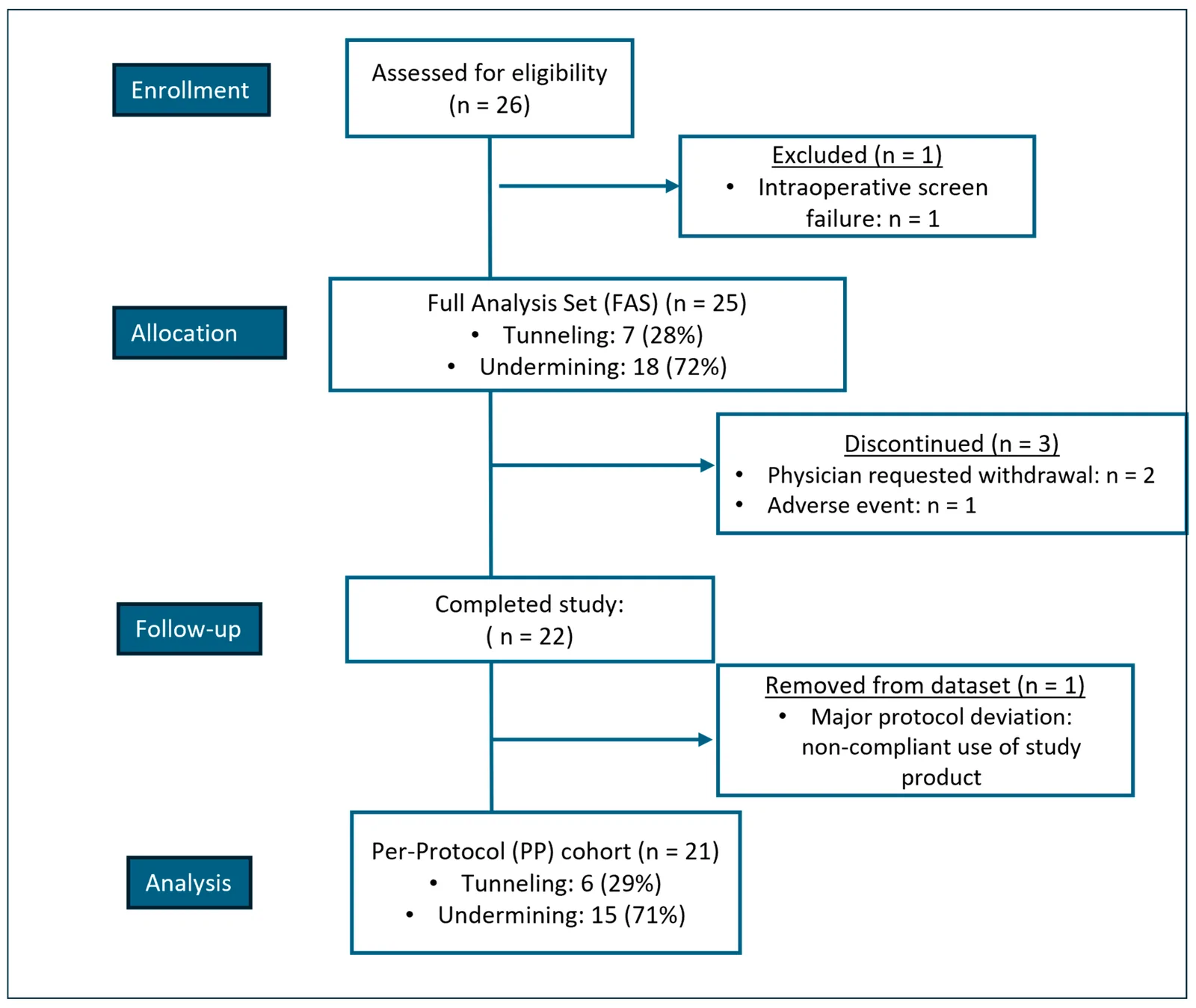
Patient enrollment, follow-up, and analysis populations.

Between December 2024 and May 2025, 26 patients were screened for eligibility. One patient was excluded for failure to meet intraoperative inclusion criteria, resulting in 25 patients enrolled in the study (Full Analysis Set [FAS]). All enrolled patients received treatment with flowable UBM applied to the undermining and tunneling aspects of their wounds and particulate and sheet UBM configurations on the surface of the wound. During the study, one patient was withdrawn for a non-product-related adverse event, and two other patients were withdrawn by the managing physician to allow for referral to comfort care and substance abuse rehabilitation facilities, respectively. Of the 22 patients who completed the study, one patient was excluded from clinical performance analysis due to a major protocol deviation (non-compliance with the protocol-defined intraoperative device application procedure). A total of 21 patients completed the study fully adherent to the study protocol (per-protocol set [PP]) (Figure 1).

The demographic information on the full study population is presented in Table 2. Of the 25 enrolled patients, the majority were male (76.0%). The average age at the time of surgery was 56.4 years ± 15.2 (range: 31–88). Patients presented with several comorbidities. The most frequently reported conditions were hypertension (32.0%), diabetes mellitus (28.0%), hyperlipidemia (20.0%), Class II obesity (20.0%), Class III obesity, and kidney disease (12.0%). Less common comorbidities include gastroesophageal reflux disease (GERD) or gastritis (8.0%) and chronic obstructive pulmonary disease (COPD, 4.0%).

The characteristics of the wounds treated in the study are summarized in Table 3. Of the enrolled patients, 7 wounds (28.0%) exhibited tunneling and 18 (72.0%) exhibited undermining. Of the 21 patients included in the per-protocol analysis, 15 (71.0%) involved undermining and 6 (29.0%) had wounds exhibiting tunneling. The per-protocol cohort consisted of 10 pressure ulcers and 11 non-pressure ulcers, with diabetic ulcers accounting for >50% of the non-pressure ulcers. Adjunctive interventions were used in three patients. One patient received revisional resection of the right foot at the site of a previous partial first ray resection, one patient was treated with an Iodosorb 1/4 in packing strip, and one patient underwent bilateral transmetatarsal amputation (TMA) revision procedures.

### 3.2. Clinical Performance Outcomes

At 12 weeks, the median percentage reduction in tunneling or undermining aspects among the 21 per-protocol population was 100.0%. A positive treatment response, defined as a decrease in aspect size, was observed in 19 of 21 wounds (90.5%). Among these 19 responding wounds, the mean ± S.D. was 84.6 ± 29.3%, with a median reduction of 100.0%. When all 21 per-protocol wounds were considered, the mean ± S.D. aspect closure was 50.4 ± 132.7%. This high variability was driven by two patients with significant wound burdens resulting from multiple pressure injuries that experienced an increase in tunneling and undermining dimensions of their index wounds during the 12-week follow-up. These wounds were statistical outliers, contributing to the wide range of observed responses (down to −501.8%) and the overall standard deviation. Given the disproportionate impact of these outliers in mean-based estimates, the median reduction may provide a more representative measure of central tendency in this small and heterogeneous cohort. Detailed results are presented in Table 4.

Table 5 shows the number of wounds by aspect type (undermining v. tunneling) and etiology (pressure v. non-pressure) that exhibited improvement or worsening trend in wound aspect at the 12-week visit. Two wounds (both pressure injuries, one tunneling and one undermining) were classified as unresponsive to treatment. Overall, 19 of 21 wounds showed improvement (size decrease) by 12 weeks, including 5 of 6 tunneling wounds and 14 of 15 undermining wounds. Improvement was observed in all non-pressure injuries (100.0%) and in most pressure injuries (80.0%).

At 12 weeks, a total of 11 wounds (52.4%) had complete resolution of tunneling and undermining aspects with a median time to resolution of 29 days (range 9–83). This included 8 of 15 undermining wounds (53.3%) and 3 of 6 tunneling wounds (50.0%).

By wound etiology, the complete aspect resolutions occurred in 9 of 11 (81.8%) non-pressure wounds and 2 of 10 (20.0%) pressure ulcer wounds (Table 6).

At 12 weeks, 7 of 21 study wounds (33.3%) closed with a median time to closure of 57 days (range 36–96). Simultaneous closure of both wound and wound aspect occurred in 5 wounds (23.8%) at a median time to closure of 57 days (range 49–78) (Table 7). No wounds in the cohort required skin grafting to achieve wound closure.

At 12 weeks, the median relative rate of wound closure was 99.3% (range −1250.0–100.0). Excluding the two non-responsive outliers, the median relative rate among the 19 responding wounds was 99.6% (range −122.2–100.0) (Table 8).

### 3.3. Safety Outcomes

Among the 25 patients who received study treatment, a total of 20 AEs were reported in 10 study participants, most of which were classified as moderate (5) or severe (4) and required hospitalization. The most frequently reported AEs were wound infection or cellulitis (6 events; 30.0%), osteomyelitis (3 events; 15.0%), and bacteremia with or without sepsis (3 events; 15.0%). Eleven serious adverse events (SAEs) were reported in 4 patients; all required hospitalization and were determined to be unrelated to the study device. The relationship of each AE to the study device was assessed by the site investigator using standard clinical causality assessment principles, including temporal relationship to device application, biologic plausibility, alternative explanations, underlying wound characteristics, and response to treatment modifications. All AEs were adjudicated by investigators as related to underlying wound pathology or comorbid conditions rather than the study device. Reported serious AEs were consistent with progression of severe underlying wound pathology and associated comorbid disease. No deaths occurred during the study period.

## 4. Discussion

This multicenter prospective study evaluated the safety and preliminary effectiveness of a flowable porcine UBM configuration for wounds complicated by tunneling or undermining features that are difficult to manage because of persistent inflammation, impaired exudate control, and complex wound geometry. Most patients demonstrated measurable reduction in tunneling or undermining dimensions over 12 weeks, and more than half achieved complete resolution of these wound aspects.

No device-related AEs were observed. Although no AEs were considered device-related, the overall AE burden was substantial, reflecting the complexity of the enrolled population. Interpretation of device safety should therefore be limited to the absence of investigator-assessed device-related complications rather than the absence of clinical adverse outcomes. These findings suggest that targeted delivery of flowable UBM may be a useful tool within a comprehensive wound care strategy for managing dead space in anatomically complex wounds.

The magnitude of aspect reduction was substantial in most patients, with a median reduction of 100% in the per-protocol cohort and similar outcomes between tunneling and undermining wounds. Outcomes varied by etiology, however, with higher rates of aspect closure in non-pressure wounds compared with pressure injuries. This difference is consistent with the prior wound-healing literature demonstrating that pressure ulcers are influenced by ongoing ischemia, mechanical stress, and systemic comorbidities that limit tissue repair. Variability in overall wound volume change also reflected the heterogeneity of wound size, etiology, and patient comorbidity, which is expected in complex wound populations.

These findings align with prior case series and retrospective reports describing favorable outcomes with extracellular matrix products in complex wounds, including those with irregular contours or deep cavities [2,3,24,25,26,27,28,29,30,31]. Flowable UBM may facilitate granulation and closure by filling dead space, modulating the inflammatory wound environment, and supporting cellular infiltration [2,3,4]. While the current study cannot isolate the mechanistic contribution of UBM, the consistent reduction in tunneling and undermining dimensions across multiple centers suggests that targeted delivery of extracellular matrix scaffolds may address a clinically important gap in wound management.

From a clinical perspective, tunneling and undermining wounds often require repeated packing, surgical unroofing, or prolonged negative-pressure therapy, all of which carry limitations related to patient discomfort, tissue loss, or cost. A flowable matrix that can be delivered into complex wound geometry may provide an additional tool for surgeons and wound care teams, particularly in patients with significant comorbidity or limited reconstructive options. Future studies should help define which wound types benefit most from application of extracellular matrix (ECM) products like Flowable UBM, optimal timing of application, and how this approach compares with existing strategies such as NPWT instillation or operative debridement. Recent evidence evaluating biologically derived extracellular matrices has reported encouraging outcomes across a range of complex wound types, supporting the potential role of scaffolds in promoting tissue repair and modulating wound healing processes. A recent systematic review by El Araby et al. further highlights the growing clinical interest in extracellular matrix-based approaches for challenging wounds and burns [33]. Although individual products differ substantially in source material, composition, and application technique, the accumulating body of evidence warrants continued investigation of biologically derived extracellular matrix therapies for anatomically complex wounds.

Importantly, this study is intentionally designed around short-term endpoints (12 weeks) to evaluate early closure potential, recognizing that complete clinical closure frequently occurs beyond this timeframe, particularly in real-world patient populations. In diabetic foot ulcer (DFU) randomized controlled trials, primary endpoints are most often assessed at 12–16 weeks, with a smaller number extending to 20–24 weeks for more severe wounds (Wagner grade 3–4). Across these studies, approximately 30–50% of ulcers achieve closure by 12 weeks under standard of care (SOC), with higher rates reported for select advanced therapies [34,35].

For pressure injuries, high-quality randomized trials more often focus on prevention rather than healing. When healing is evaluated, timelines vary substantially by stage: stage II injuries may resolve within weeks to a few months, whereas stage III–IV injuries frequently require several months to over one year to achieve closure, particularly in older or immobile patient populations. Real-world observational data further demonstrate that advanced pressure injuries commonly exceed the duration of typical clinical trials, with persistent non-closure despite standard therapy and even modern adjunctive treatments. Accordingly, a 12-week endpoint is insufficient to fully characterize healing in moderate to severe pressure injuries and is best viewed as reflecting early healing trajectory rather than a definitive outcome [36,37].

Certain limitations merit consideration in this study. The study should be considered exploratory in nature and was not powered to detect statistically significant treatment effects. The single-arm design precludes determination of causality and prevents isolation of the independent effect of flowable UBM from concurrent wound care interventions. Consequently, improvements observed during the study should be interpreted as clinical outcomes observed in patients managed with a comprehensive wound care regimen that included flowable UBM, rather than evidence of comparative efficacy. The sample size was small, wound etiologies were heterogeneous, and wound measurements were performed manually, introducing potential measurement variability. Although wound assessments were conducted by trained wound care clinicians using standardized site procedures, manual measurement of complex three-dimensional tunneling features remains subject to inter-rater variability and may not fully capture changes in wound geometry. Future studies incorporating advanced imaging technologies and objective three-dimensional wound assessment methods may further improve measurement consistency and precision. Follow-up was limited to 12 weeks, which may not fully capture the longer-term healing trajectory of chronic wounds. Larger controlled studies with standardized co-interventions, longer follow-up, and patient-reported outcome measures are needed to better define comparative effectiveness.

Despite the above-mentioned limitations, this study provides prospective multicenter evidence focused specifically on wounds complicated by tunneling or undermining, a subset of complex wounds for which standardized management approaches remain limited. These findings suggest that targeted delivery of flowable UBM may be a useful adjunct within comprehensive wound care; however, controlled studies are required before conclusions regarding comparative effectiveness can be made.

## 5. Conclusions

Tunneling and undermining are particularly challenging wound characteristics associated with persistent inflammation, increased infection risk, and delayed healing [2,3,4]. By creating areas of dead space and complex wound geometry, these features limit effective delivery of standard therapies and complicate wound management. In many clinical settings, standardized or practical strategies for addressing tunneling and undermining remain limited, highlighting an important unmet need.

This study provides prospective, multi-center preliminary data on the use of flowable UBM configuration in wounds complicated by tunneling or undermining. In a diverse patient population with significant comorbidities, treatment was associated with meaningful reduction, and in many cases, complete resolution of these complex wound features. These findings suggest that targeted delivery of flowable UBM may help manage dead space and support healing in anatomically challenging wounds. Although larger controlled studies are required to confirm these observations and define optimal patient selection, this work adds to the limited evidence base for managing tunneled or undermined wounds and supports consideration of flowable UBM as an adjunctive strategy within comprehensive wound care.

## Figures and Tables

**Table 1:** Inclusion and exclusion criteria for patient selection.

Inclusion	Exclusion
Screening Eligibility Criteria	
Patient is ≥22 years at the time of consent Patient with a diagnosis of pressure injury, venous ulcer, diabetic ulcer, necrotizing soft tissue infection-associated wound, dehiscing and/or draining wounds associated with open abdominal procedures, or any indication per the Instructions for Use Patient is willing and able to adhere to protocol requirements and comply with the study follow-up regimen Patient has reviewed the IRB/IEC-approved consent form, has been properly consented per the protocol and has documented their consent to participate in the study by signing the IRB/IEC-approved consent form	Burn as etiology of wound Unmanaged infection and/or osteomyelitis as determined pre-operatively Known allergy, hypersensitivity, or objection to porcine materials, due to religion, culture, or other Patient report of concurrent participation in another clinical study that would interfere with this study Any condition, concomitant medication, or concomitant treatment that, in the opinion of the investigator, would preclude the use of the study device or preclude the patient from completing the follow-up requirements
**Intraoperative Eligibility Criteria**	
Tunneling aspect of wound ≥ 5 cm deep or undermined aspect of wound > 2 cm deep post-debridement If there is more than one wound, the wound selected as the study assessment wound should be separated by ≥2 cm post-debridement No further debridement or surgical intervention is necessary to remove necrotic tissue from wound bed prior to index procedure	Wounds ≥ 500 cm^2^ post-debridement Unmanaged infection and/or osteomyelitis

Shaded rows indicate major eligibility assessment stages (screening and intraoperative criteria).

**Table 2:** Patient demographics and baseline characteristics.

Parameter	FAS (N = 25)
Sex, n (%)	
Male	19 (76.0)
Female	6 (24.0)
Race, n (%)	
Asian	3 (12.0)
Black or African American	11 (44.0)
Caucasian	10 (40.0)
Other	1 (4.0)
Age (years) at time of surgery: mean ± SD (range)	56.4 ± 15.2 (31, 88)
Height (In): mean ± SD (range)	68.4 ± 4.2 (61.0, 77.0)
Weight (lbs.): mean ± SD (range)	191.3 ± 63.6 (97.0, 360.0)
Alcohol use, n (%)	
Current	3 (12.0)
Former	6 (24.0)
Never	16 (64.0)
Nicotine use, n (%)	
Current	4 (16.0)
Former	8 (32.0)
Never	13 (52.0)
Concomitant treatment, n (%)	
None	21 (84.0)
1	3 (12.0)
2	1 (4.0)
Comorbidities, n (%)	
Hypertension	8 (32.0)
Diabetes mellitus (Types I & II)	7 (28.0)
Hyperlipidemia	5 (20.0)
Acute & chronic kidney disease	3 (12.0)
Moderate- severe obesity (BMI 30–40)	5 (20.0)
Morbid obesity (BMI > 40)	3 (12.0)
GERD/Gastritis	2 (8.0)
COPD	1 (4.0)

**Table 3:** Study wound characteristics.

Parameter	FAS (N = 25) n (%)	PP (N = 21) n (%)
Wound type		
Pressure ulcers	11 (44.0)	10 (48.0)
Non-pressure ulcers	14 (56.0)	11 (52.0)
Diabetic ulcer	6 (24.0)	6 (28.6)
NSTI	1 (4.0)	1 (4.8)
Open abdominal wound	1 (4.0)	0
Other	6 (24.0)	4 (19.0)
Cellulitis	1 (4.0)	1 (4.8)
Dog bite	1 (4.0)	0
Draining wound post-fall	1 (4.0)	0
Post-hematoma evacuation	1 (4.0)	1 (4.8)
Post-incision & post-drainage for infected hematoma	1 (4.0)	1 (4.8)
Right knee post-surgical dehiscence	1 (4.0)	1 (4.8)
Wound features		
Tunneling	7 (28.0)	6 (29.0)
Undermining	18 (72.0)	15 (71.0)

**Table 4:** Relative rate of closure of tunneling or undermining aspects at 12 weeks.

Parameter	PP	Treatment Responders
n	21	19
Mean ± SD (range)	50.4 ± 132.7 (−501.8, 100.0)	84.6 ± 29.3 (13.3, 100.0)
Median	100.0	100.0

**Table 5:** Trend of wound closure at 12 weeks.

Parameter	PP (N = 21) n (%)
Aspect type	
Tunneling	
n	6 (100.0)
Negative (worsened)	1 (16.7)
Positive (improved)	5 (83.3)
Undermining	
n	15 (100.0)
Negative (worsened)	1 (6.7)
Positive (improved)	14 (93.3)
Etiology	
Pressure injury	
n	10 (100.0)
Negative (worsened)	2 (20.0)
Positive (improved)	8 (80.0)
Non-pressure injury	
n	11 (100.0)
Negative (worsened)	NA
Positive (improved)	11 (100.0)

**Table 6:** Count (percentage) of complete resolution of aspect at 12 weeks.

Parameter	PP (N = 21) n (%)
Achieved aspect closure	11 (52.4)
Achieved surface wound closure	7 (33.3)
Achieved full (surface + aspect) wound closure	5 (23.8)
Aspect type	
Tunneling wounds (n = 6)	
Achieved aspect closure	3 (50.0)
Achieved surface wound closure	2 (33.3)
Achieved full wound closure	2 (33.3)
Undermining wounds (n = 15)	
Achieved aspect closure	8 (53.3)
Achieved surface wound closure	5 (33.3)
Achieved full wound closure	3 (20.0)
Etiology	
Pressure injury wounds (n = 10)	
Achieved aspect closure	2 (20.0)
Achieved surface wound closure	3 (30.0)
Achieved full wound closure	1 (10.0)
Non-pressure injury wounds (n = 11)	
Achieved aspect closure	9 (81.8)
Achieved surface wound closure	4 (36.4)
Achieved full wound closure	4 (36.4)

**Table 7:** Time to wound closure outcomes.

Parameter	PP (N = 21)
Time to aspect (undermining/tunneling) closure (days)	
n (achieved aspect closure)	11
Median	29.0
Mean ± SD	43.5 ± 25.3
Range	9, 83
Time to surface wound closure (days)	
n (achieved surface wound closure)	7
Median	57.0
Mean ± SD	61.4 ± 20.1
Range	36, 96
Time to full (surface + aspect) wound closure (days)	
n (achieved full wound closure)	5
Median	57.0
Mean ± SD	59.6 ± 11.9
Range	49, 78

**Table 8:** Relative rate of wound closure at 12 weeks.

Parameter	PP	Treatment Responders
n	21	19
Median	99.3%	99.6%
Mean ± SD (range)	−55.9 ± 397.2 (−1250.0, 100.0)	68.6 ± 58.1 (−122.2, 100.0)

## Data Availability

All relevant data supporting reported results of this study is made available with this manuscript. Any additional data that may be required by individuals or organizations will be made available upon request.

## Appendix A

For tunneling wound aspects, volume was used to calculate the relative rate of closure:

Volume of a tunnel:

(A1) $$V_{Tunnel}=\frac{1}{12}\pi d_{t}^{2}l$$

where d_t_ = tunneling diameter and l = tunneling length (or depth).

Then, the relative rate of the tunneling aspect closure was calculated at each time, n, as follows:

(A2) $$Tunnel\;\%\;closure\;relative\;rate\;at\;given\;visit\;n=\frac{\frac{1}{12}\pi d_{t_{0}}^{2}l_{0}-\frac{1}{12}\pi d_{t_{n}}^{2}l_{n}}{\frac{1}{12}\pi d_{t_{0}}^{2}l_{0}}*100\%$$

The tunnel volume closure reduction rate at time *n* was calculated as follows:

(A3) $$\frac{\left[\left(\frac{\frac{1}{12}\pi d_{t_{0}}^{2}l_{0}-\frac{1}{12}\pi d_{t_{n}}^{2}l_{n}}{\frac{1}{12}\pi d_{t_{0}}^{2}l_{0}}\right)*100\%\right]}{time\;(weeks\;total)}$$

For undermined wound aspects, depth was used to calculate the relative rate of closure. The diameter of the undermined aspect was not measured.

Undermining % closure relative rate at giving visit n:

(A4) $$\left(\frac{l_{0}-l_{n}}{l_{0}}\right)*100\%$$

Undermining % closure reduction rate at time *n*:

(A5) $$\frac{\left[\left(\frac{l_{0}-l_{n}}{l_{0}}\right)*100\%\right]}{time\;(weeks\;total)}$$

Formulas (A2) and (A4) were used to calculate the relative rate of closure, where initial volume or depth were those measured on treatment visit 1 (day 0) and the final volume or depth were those measured at the week-12 visit.

Secondary endpoints included:

- Relative rate of closure of a tunneling or undermining feature as determined manually, through measurement of opening size and depth of managed wound at each visit, relative to first visit/application post-debridement.
